# Supplementary material for: The Ca2+ influx through the mammalian skeletal muscle dihydropyridine receptor is irrelevant for muscle performance
Source: Nat Commun. 2017 Sep 7;8:475. doi: 10.1038/s41467-017-00629-x (PMC5589907; doi:10.1038/s41467-017-00629-x)
Supplement: Supplementary file 1 — Supplementary Information [file 41467_2017_629_MOESM1_ESM.pdf]

Description of Supplementary Files

File name: Supplementary Information
Description: Supplementary figures and supplementary tables.

File name: Peer review file

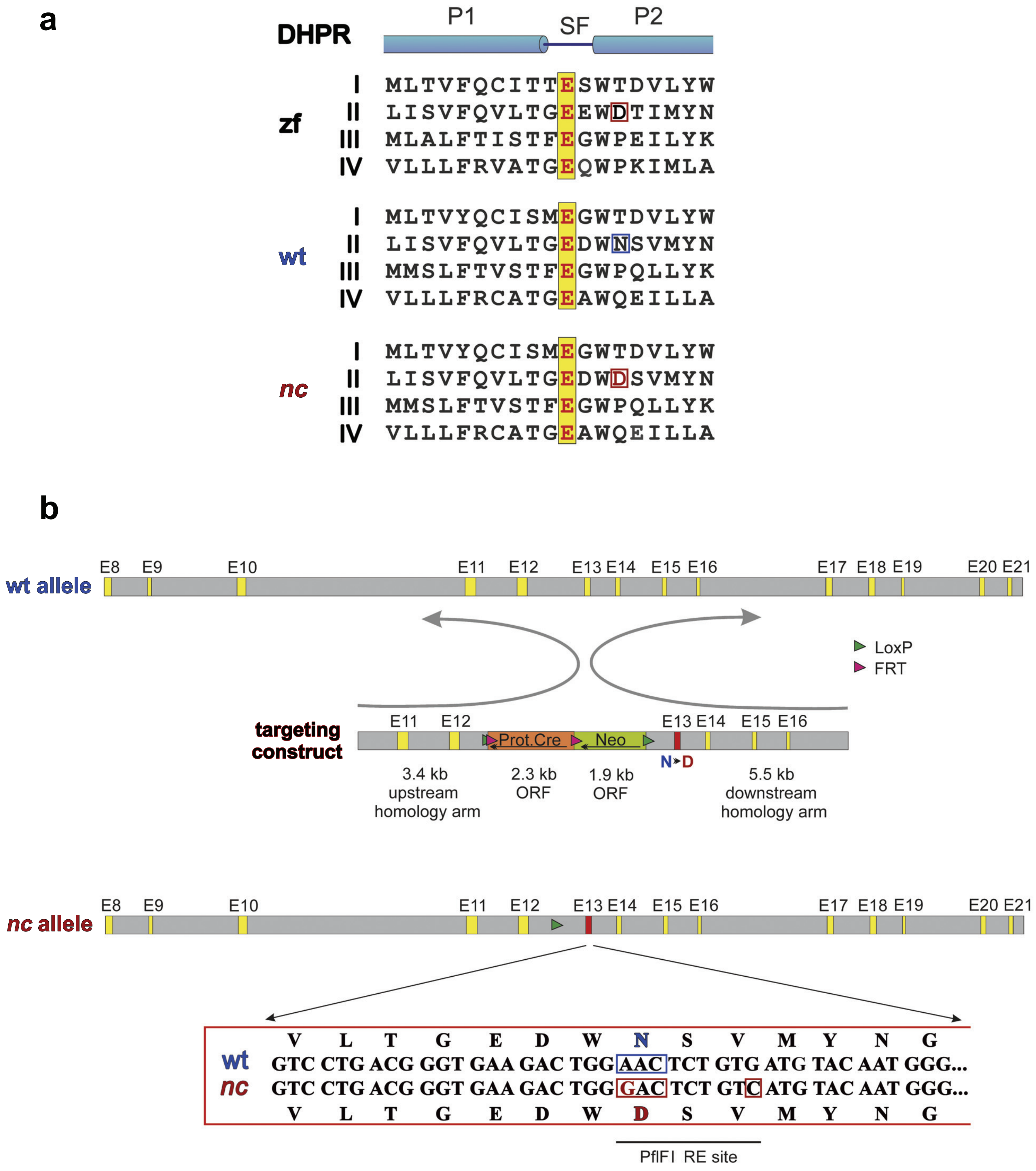

Supplementary Figure 1 | Generation of the non-conducting (*nc*) DHPR knock-in mouse model. (**a**) Sequence alignment of residues forming and framing the skeletal muscle DHPRα_{1S} Ca^{2+} selectivity filter of zebrafish (zf), wild-type mouse (wt), and the non-Ca^{2+}-conducting *nc*DHPR mouse model (*nc*). Homologous repeats I-IV are aligned and the critical EEEE residues of the Ca^{2+} selectivity filter are *boxed in yellow*. Introduction of the zebrafish pore loop II residue D617 (*red box*) into the corresponding position N617 (*blue box*) in mouse, yielded the N617D mutant *nc*DHPR mouse model (*red box*). The *blue bar* indicates the position of the two pore helices P1 and P2 and the *blue line* depicts the selectivity filter vestibule (SF)[70]. (**b**) Schematic representation of the targeting construct used to generate the *nc*DHPR mouse model. For engineering the targeting construct, codon 8 in exon 13 of the mouse *CACNA1S* gene was mutated from AAC (*blue box*) to GAC (*red box*), coding for D instead of N. For genotype confirmation by RFLP, an additional silent mutation was introduced in codon 10 (GTG to GTC), to generate a PflF1 restriction enzyme (RE) site. Modified exon 13 was flanked upstream by floxed neomycin (*Neo*) and protamine-Cre (*Prot. Cre*) cassettes, inserted in antisense direction. This assembly allows auto-excision of the floxed cassettes in the male germ line but not in embryonic stem cells of the knock-in mice.

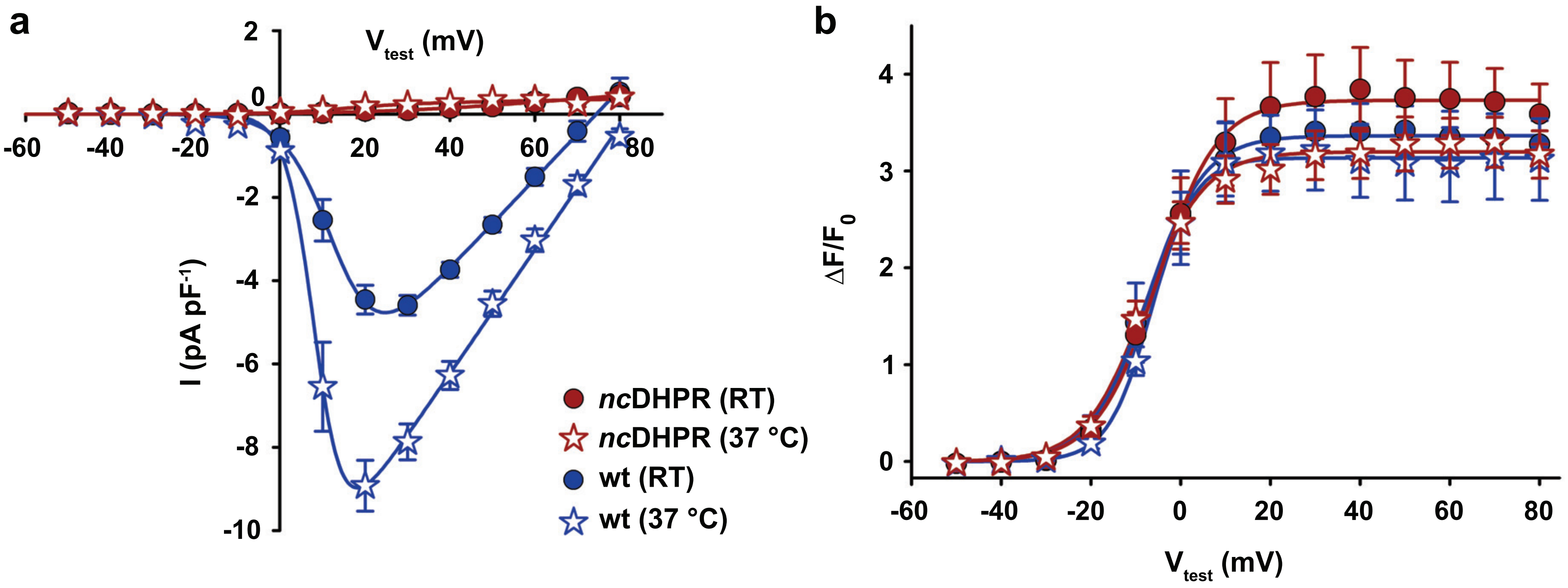

Supplementary Figure 2 | The *nc*DHPR mouse completely lacks DHPR Ca^{2+} influx even under current amplifying conditions. Voltage-dependence of DHPR-mediated Ca^{2+} currents and SR Ca^{2+} release at room temperature and physiological temperature (37 °C). (**a**) The magnitude of DHPR Ca^{2+} currents recorded in wt myotubes was doubled at 37 °C (n=20) compared to ($P<0.001$) room temperature (RT) (n=7). Conversely, even at 37 °C no DHPR Ca^{2+} currents could be evoked in the *nc*DHPR myotubes (n=22). (**b**) Voltage-dependence of maximal Ca^{2+} release is unaltered ($P>0.05$) in *nc*DHPR ($(\Delta F/F0)_{max}$=3.22±0.25); n=16) compared to wt ($(\Delta F/F_0)_{max}$=3.13±0.39; n=15) myotubes and thus does not show temperature dependence. Data are represented as mean±s.e.m.; *P* determined by unpaired Student's *t*-test.

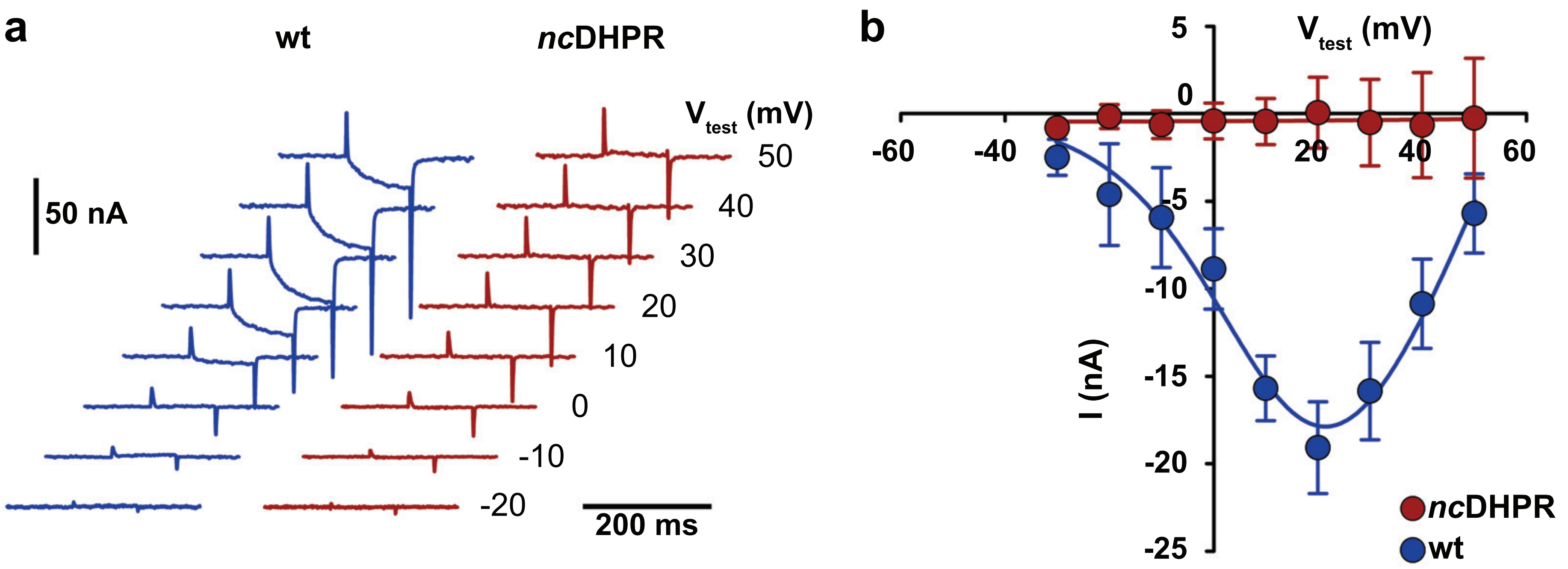

Supplementary Figure 3 | Absence of DHPR inward Ca^{2+} current in adult toe fibres of *nc*DHPR mice. (**a**) Representative two-electrode voltage clamp recordings from isolated toe fibres (*musculus interosseus*) indicating the complete loss of DHPR Ca^{2+} influx in *nc*DHPR mice compared to ($P<0.001$) wt mice. In contrast, wt fibres showed typical slowly activating DHPR-mediated inward Ca^{2+} currents. Scale bars, 200 ms (horizontal), 50 nA (vertical). (**b**) Current-voltage relationship for DHPR Ca^{2+} currents recorded from *nc*DHPR (n=17) and wt (n=16) fibres. Data are represented as mean±s.e.m.; *P* determined by unpaired Student's *t*-test.

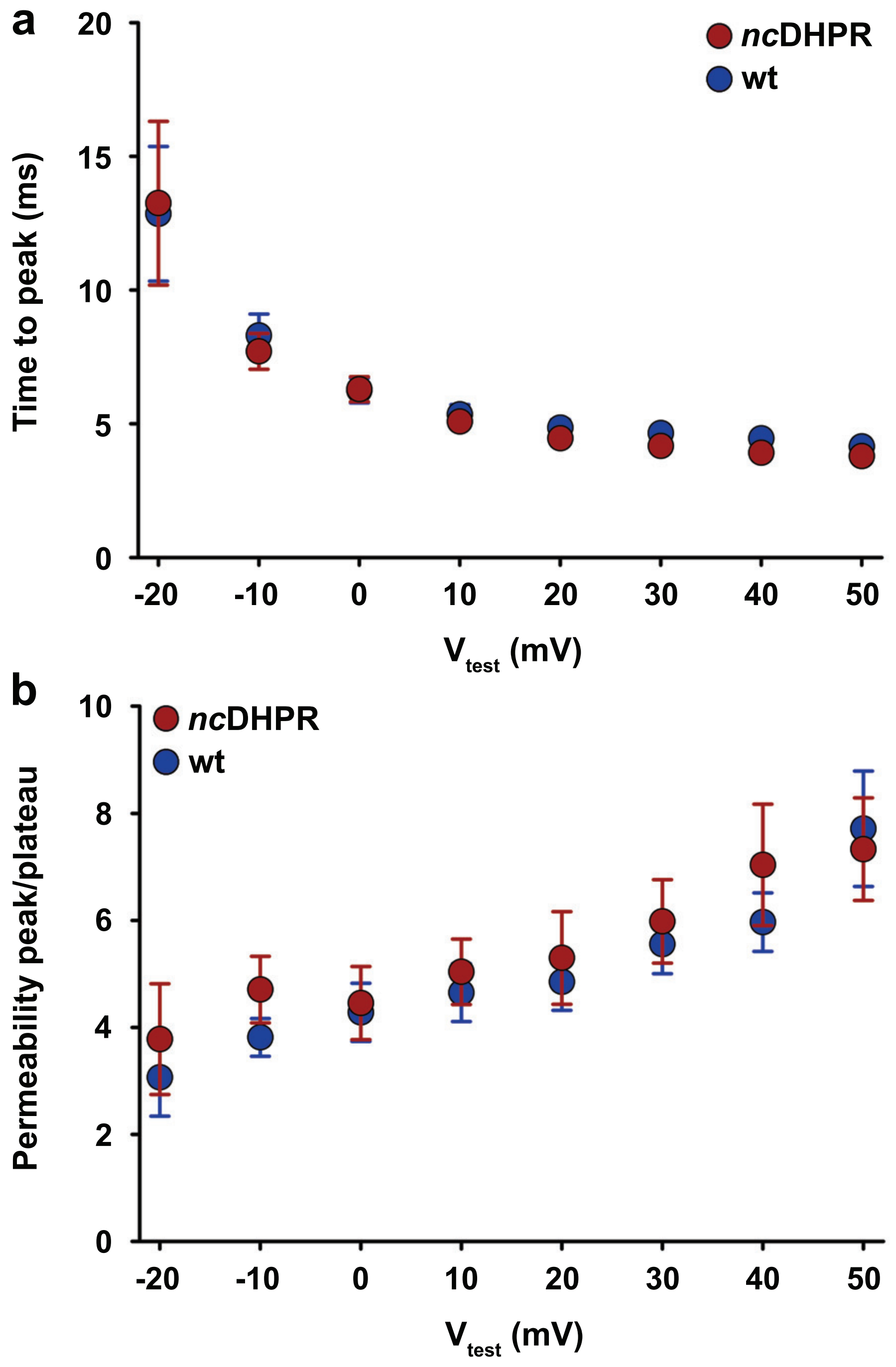

Supplementary Figure 4 | Kinetics of depolarization-activated SR Ca^{2+} release is unaltered in *nc*DHPR mice. No difference (*P*>0.05) was observed in voltage dependence of (**a**) the rising time to peak and (**b**) ratio of permeability peak to plateau between *nc*DHPR (n=12) and wt (n=10) fibres. Data are represented as mean±s.e.m.; *P* determined by unpaired Student's *t*-test.

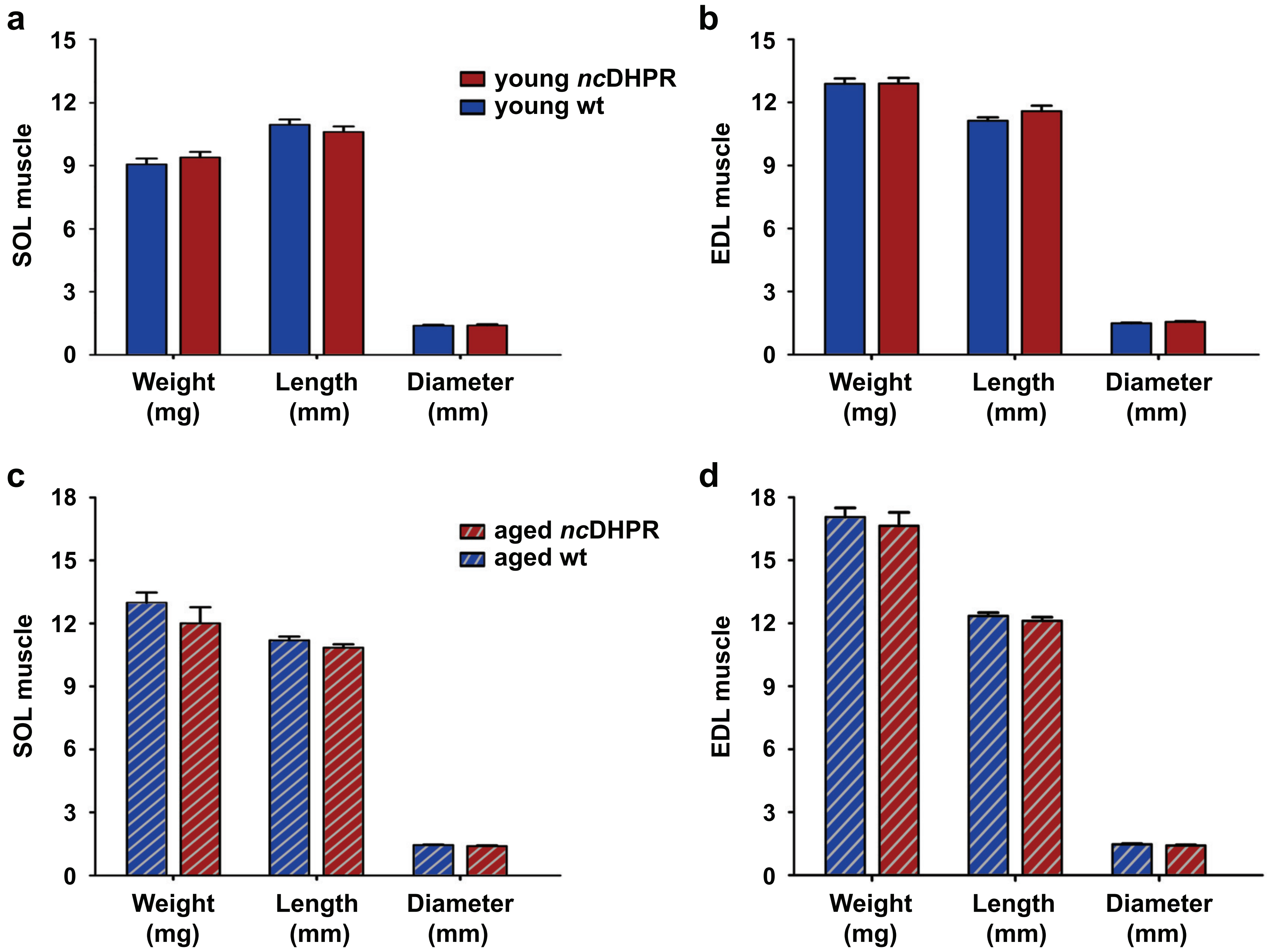

Supplementary Figure 5 | Physical muscle parameters are unaltered in *nc*DHPR mice. No differences (P>0.05) in mean wet weight, length, and diameter were observed in SOL (**a,c**) and EDL (**b,d**) muscles isolated from either young (**a,b**) *nc*DHPR (SOL, n=12; EDL, n=19) and wt (SOL, n=9; EDL, n=18) mice or aged (**c,e**) *nc*DHPR (SOL, n=19; EDL, n=20) and wt (SOL, n=18; EDL, n=22) mice. Bars represent mean±s.e.m.; P determined by unpaired Student's t-test.

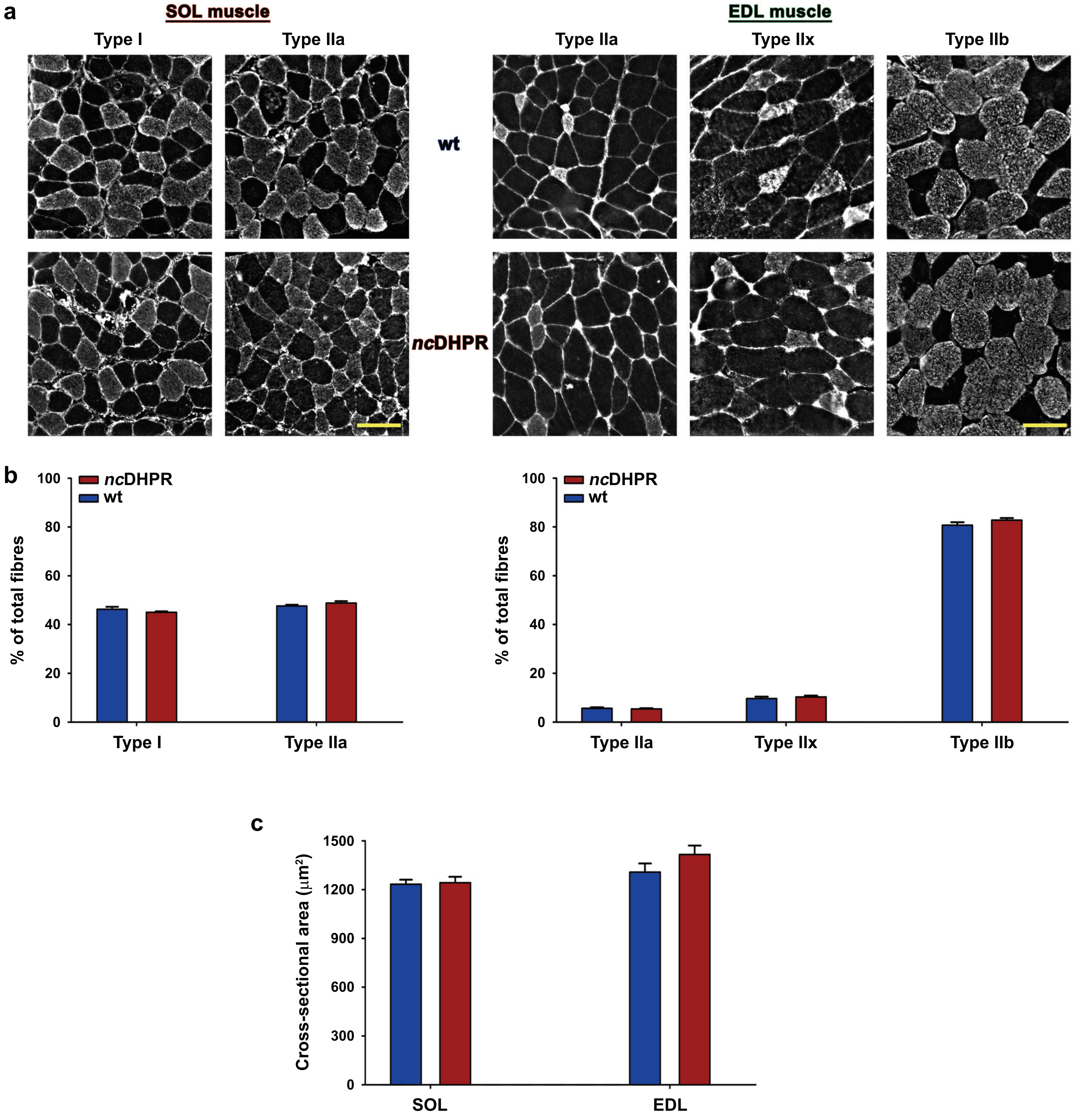

Supplementary Figure 6 | Muscle fibre-type composition and CSA are unaltered in *nc*DHPR mice. **(a)** Representative images of transverse sections of SOL and EDL muscles from 2-3 months-old *nc*DHPR and wt mice, immunostained with fibre-type specific myosin heavy chain antibodies. Scale bars, 50 µm. (**b**) Fractional distribution of fibre-types in SOL and EDL muscles of *nc*DHPR and wt mice indicates comparable (P>0.05) muscle composition regarding Type I (slow), Type IIa (moderately fast), Type IIx (fast), and Type IIb fibres (very fast) between the two genotypes. Number of fibres counted in SOL - Type I: 4,386 (*nc*DHPR), 4,170 (wt); Type IIa: 5,117 (*nc*DHPR), 2,565 (wt). Number of fibres counted in EDL - Type IIa: 4,301 (*nc*DHPR), 3,870 (wt); Type IIb: 3,573 (*nc*DHPR), 4,210 (wt); Type IIx: 4,619 (*nc*DHPR), 2,933 (wt). (**c**) Average fibre CSA of SOL and EDL muscles were indistinguishable (P>0.05) between *nc*DHPR (SOL: 1,242±36 µm^2; EDL: 1,415±55 µm^2) and wt (SOL: 1,233±27 µm^2; EDL: 1,307±53 µm^2) mice (n=110 fibres for both muscle types and both genotypes). Bars represent mean±s.e.m.; P determined by unpaired Student's t-test.

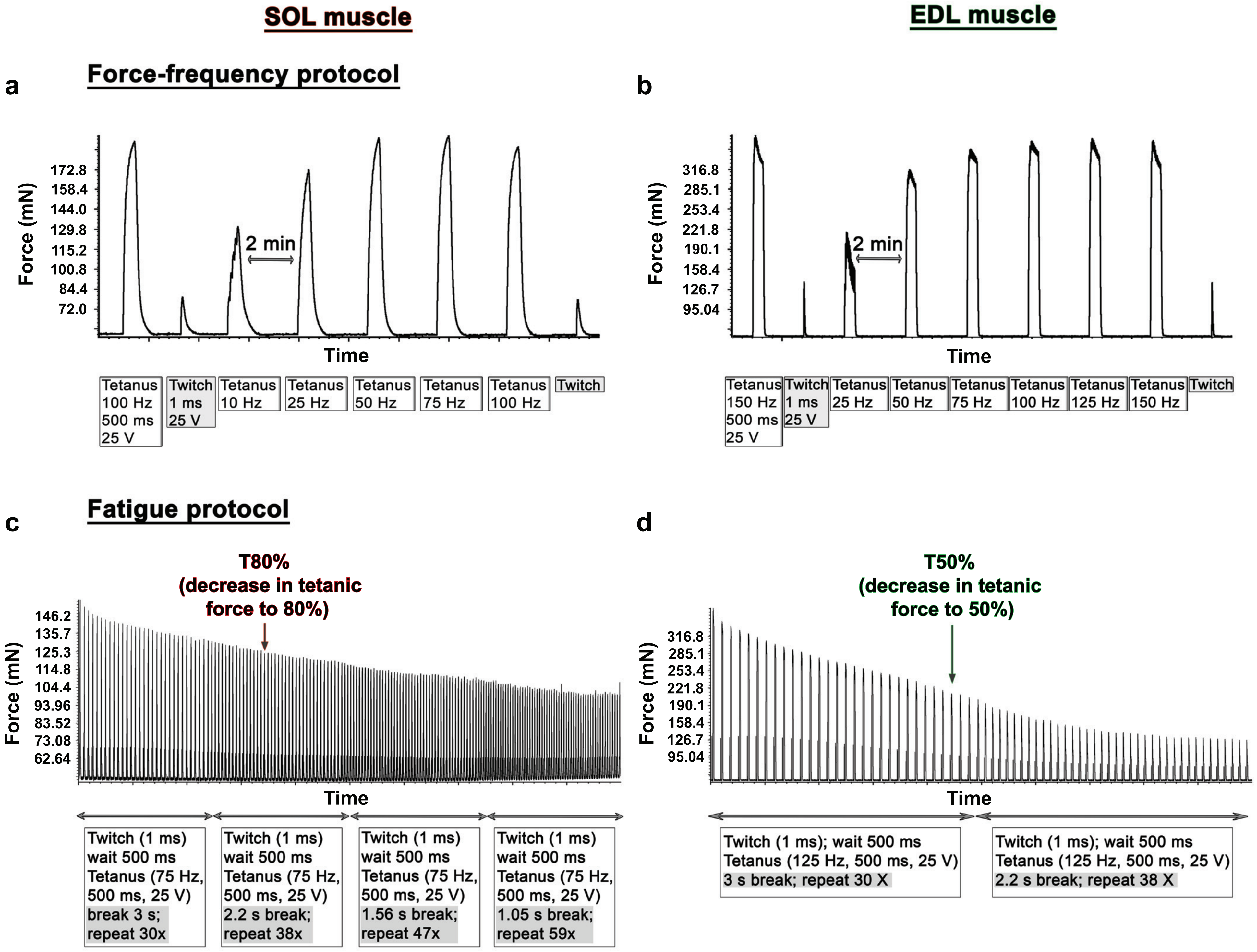

Supplementary Figure 7 | *Ex vivo* isometric contraction protocols. (**a**,**b**) Representative force-frequency recordings from isolated adult *nc*DHPR (**a**) SOL and (**b**) EDL muscles at increasing stimulation frequencies with fixed 2-min recovery interval. (**c**,**d**) Representative fatigue recordings from (**c**) SOL and (**d**) EDL muscles elicited during repetitive high frequency tetanic stimulations with fixed stimulation frequency but decreasing recovery breaks (*marked in grey*) every 2 min. Decrease in tetanic force to 80% (T80% in case of SOL) and 50% (T50% in case of EDL) is indicated. All recordings were performed at room temperature (~26 °C). The stimulation parameters are indicated below the x-axes for all the representative recordings.

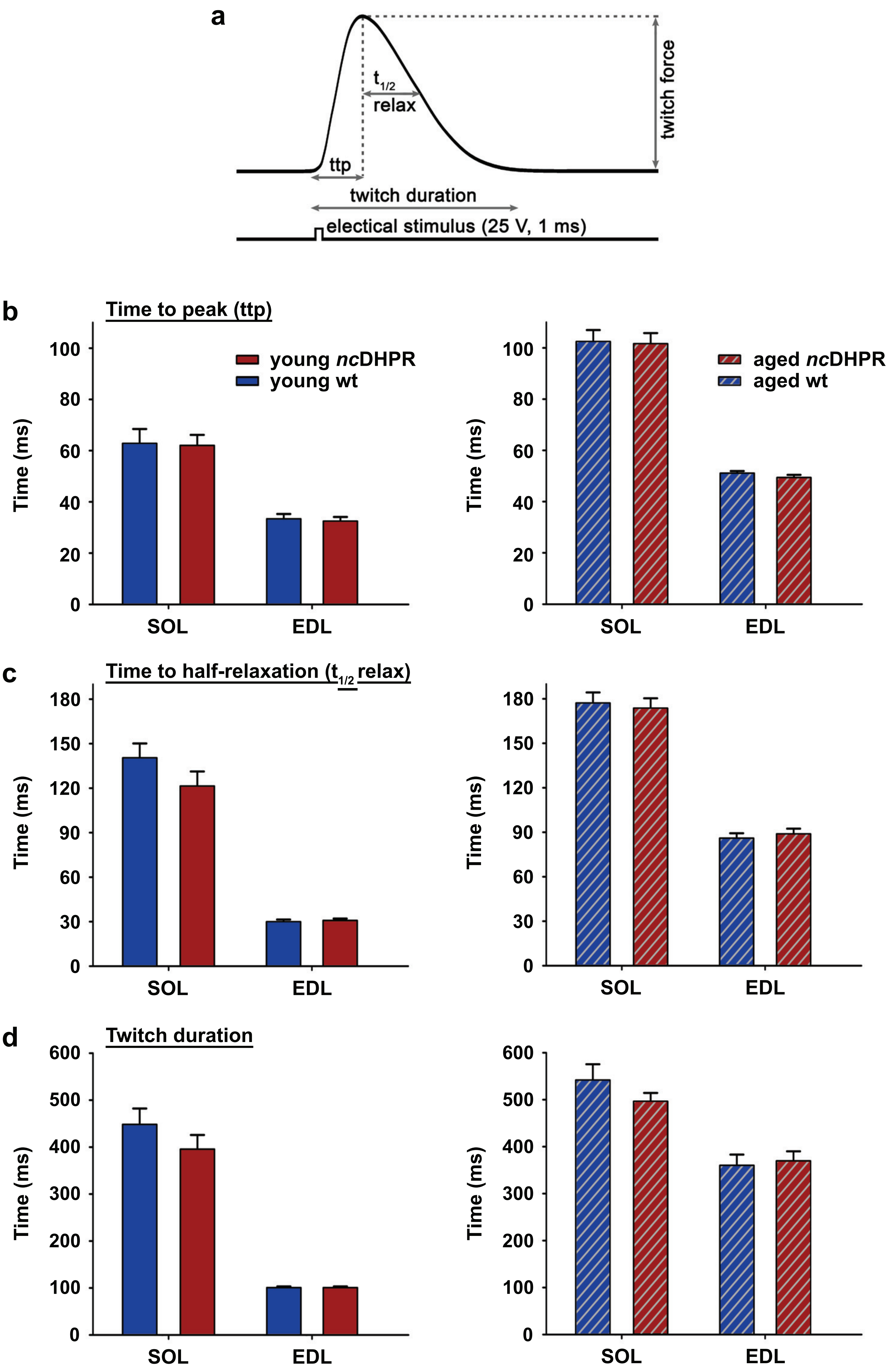

Supplementary Figure 8. | Comparison of twitch parameters between *nc*DHPR and wt muscles. (**a**) Schematic representation of the phases of a twitch contraction in response to an electrical stimulus. (**b,c,d**) Twitch contractile properties of SOL and EDL muscles isolated from young (left raphs) or a e (right graphs) mice viz. (**b**) time to peak (ttp), (**c**) time to half-relaxation ($t_{1/2}$) and (**d**) twitch duration were indistinguishable (P>0.05) between *nc*DHPR and wt mice. Experiments were performed at room temperature (~26 °C). Bars represent mean±s.e.m.; P determined by unpaired Student's t-test.

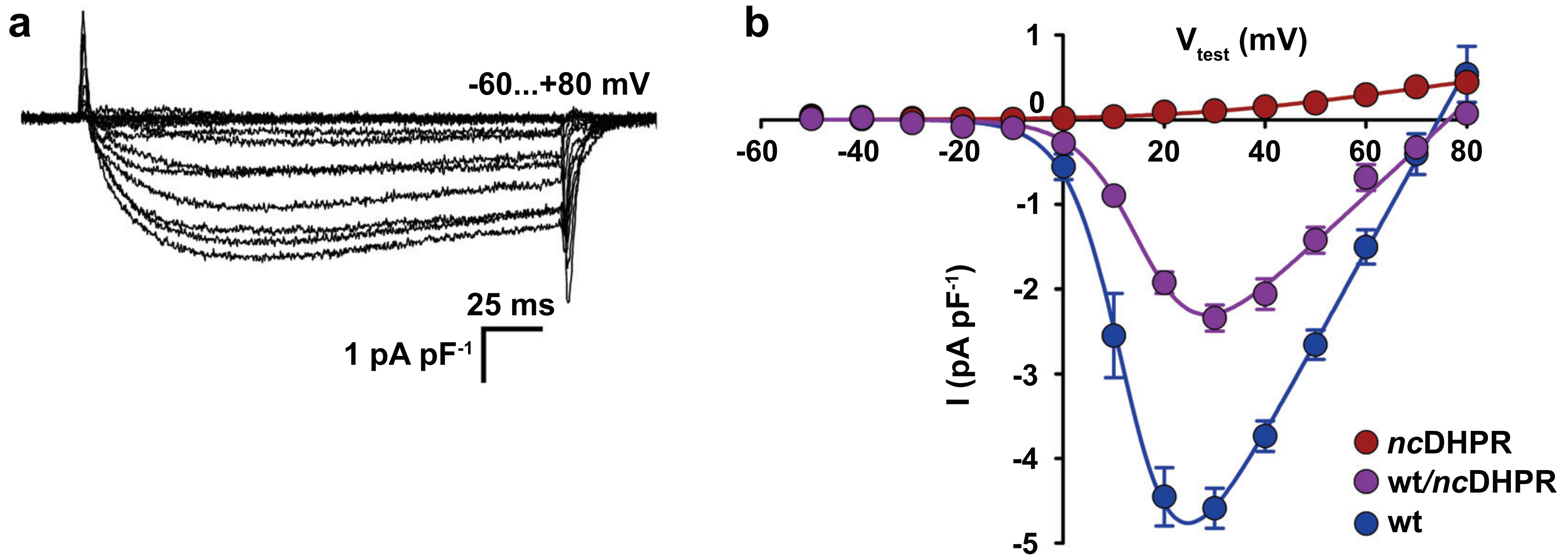

Supplementary Figure 9 | Amplitude of the DHPR Ca^{2+} current in the heterozygous wt/*nc*DHPR mouse does not point to an adaptive up-regulation of the DHPR. (**a**) Representative whole-cell inward Ca^{2+} current recording from myotubes isolated from 3-4 days old heterozygous wt/*nc*DHPR mice. Scale bars, 25 ms (horizontal), 1 pA pF^{-1} (vertical). (**b**) Current-voltage relationship of DHPR-mediated Ca^{2+} currents recorded from homozygous *nc*DHPR (n=13), heterozygous wt/*nc*DHPR (n=11) and wt (n=7) myotubes. A reduction of 51% in the amplitude of Ca^{2+} currents in heterozygous wt/*nc*DHPR myotubes compared ($P<0.001$) to wt myotubes indicates no adaptive upregulation of the DHPR in heterozygous wt/*nc*DHPR mice. Data are represented as mean±s.e.m.; *P* determined by unpaired Student's *t*-test.

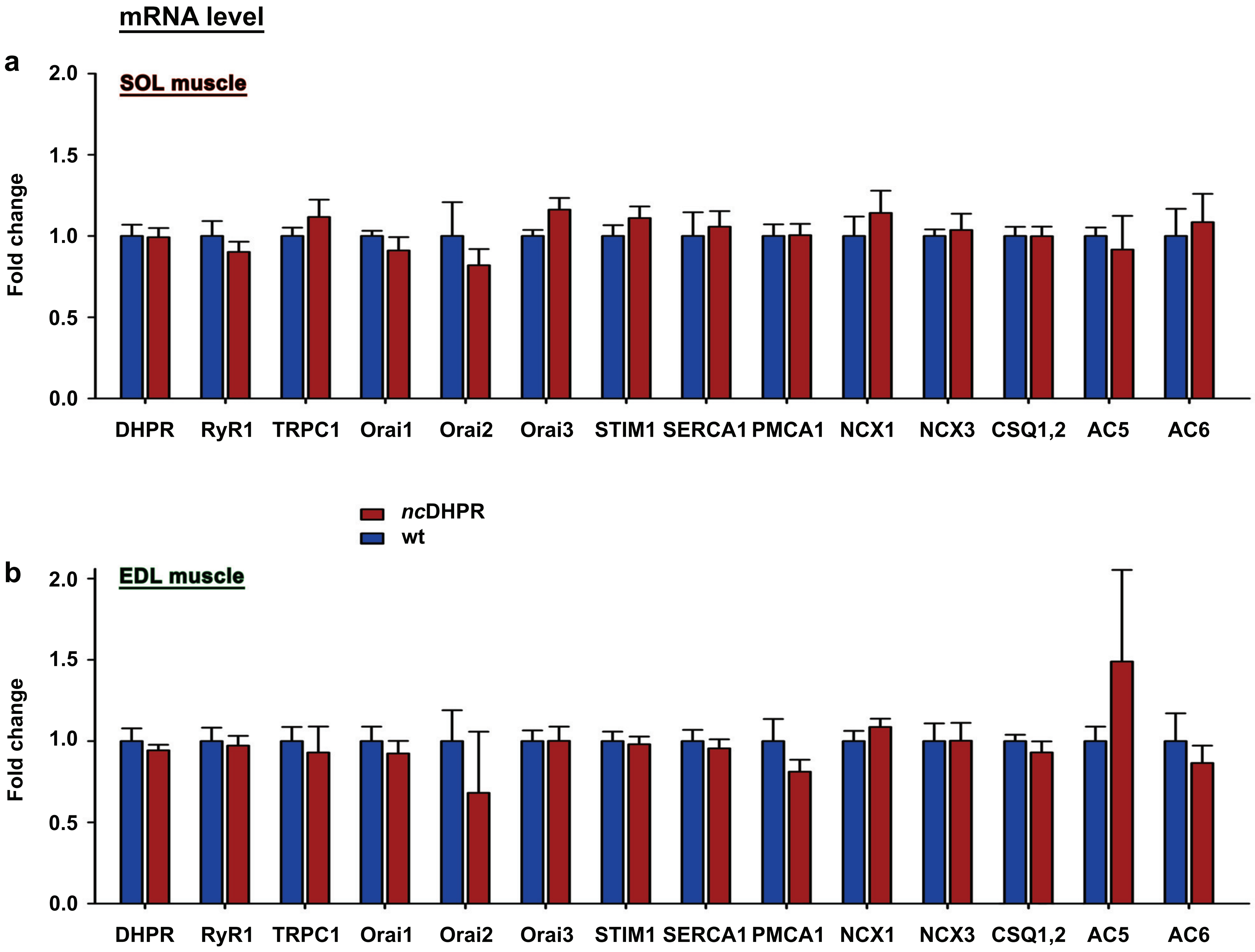

Supplementary Figure 10 | TaqMan® qRT-PCR assay of key triadic proteins involved in EC coupling and Ca^{2+} homeostasis in SOL and EDL muscles. No compensatory transcriptional regulation of crucial proteins involved in EC coupling and Ca^{2+} handling is observed in (**a**) SOL and (**b**) EDL muscles isolated from adult *nc*DHPR mice (SOL, n=6; EDL, n=5) compared to ($P>0.05$) wt counter mates (SOL and EDL, n=5). For each gene of interest, the expression level was normalized to the reference gene *EEF1A2* (Eukaryotic translation elongation factor 1 alpha 2)[68]. Bars represent mean±s.e.m.-fold change relative to wt; *P* determined by unpaired Student's *t*-test.

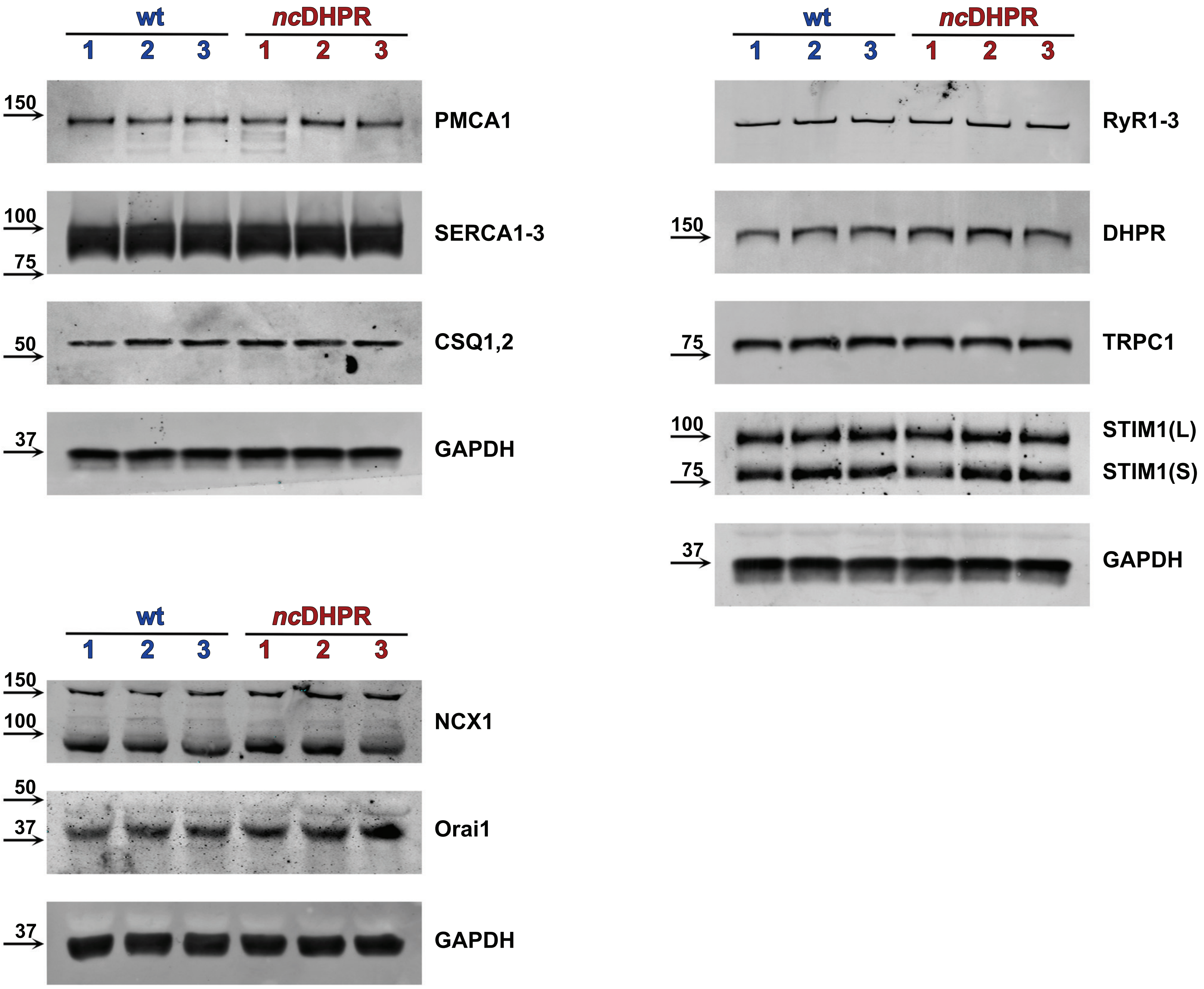

Supplementary Figure 11 | Cropped scans of immunoblots. Western blots of key triadic proteins in TA muscles from *nc*DHPR (n=3) and wt control mice (n=3). GAPDH was used as a loading control.

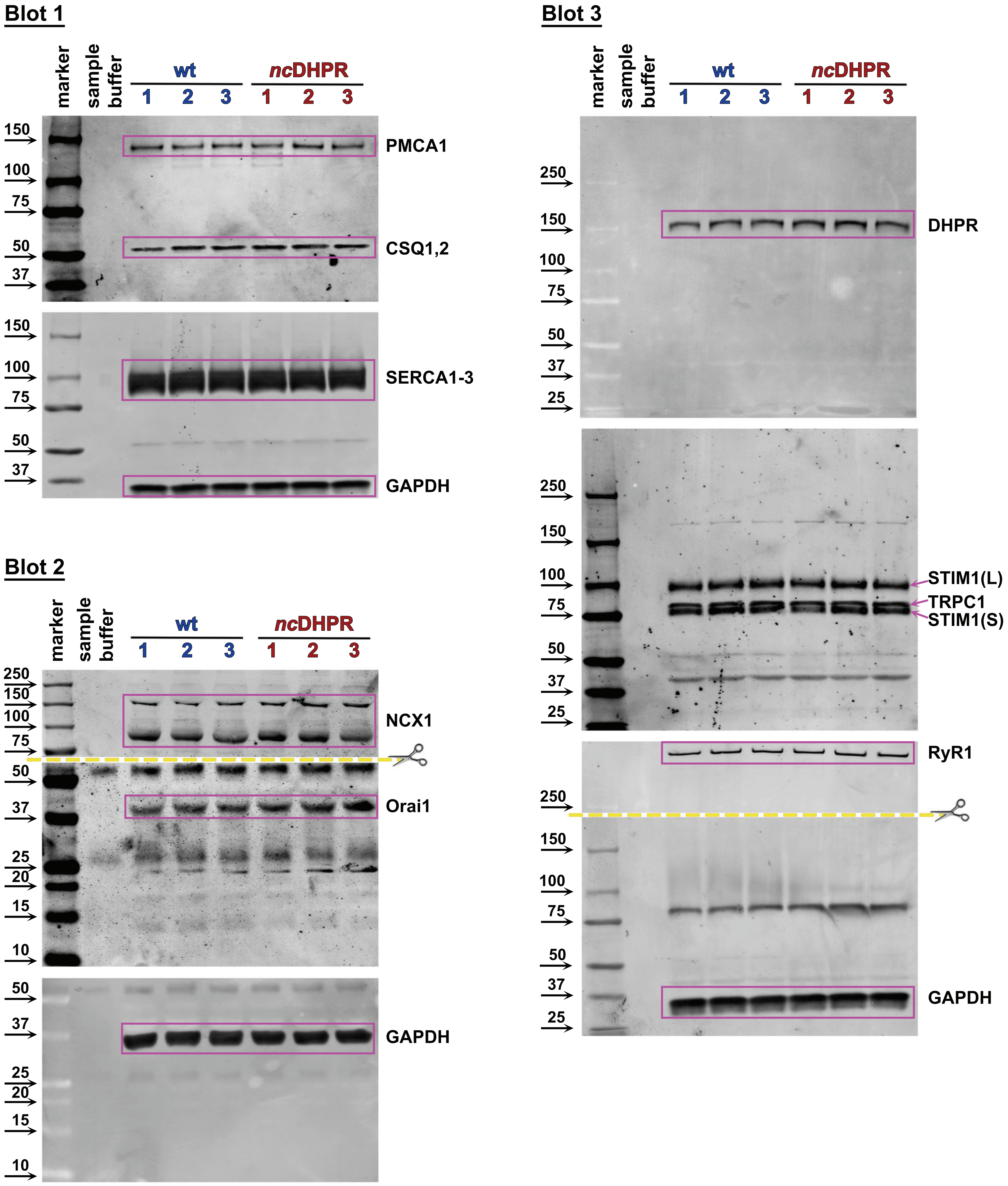

Supplementary Figure 12 | Uncropped scans of immunoblots. Magenta boxes indicate the areas used in Supplementary Fig. 11. Blots were probed with primary antibodies as specified. Subsequently, same blots were either cut (yellow dotted line) or entire blots were re-probed with different primary antibodies. GAPDH was used as a loading control on every blot.

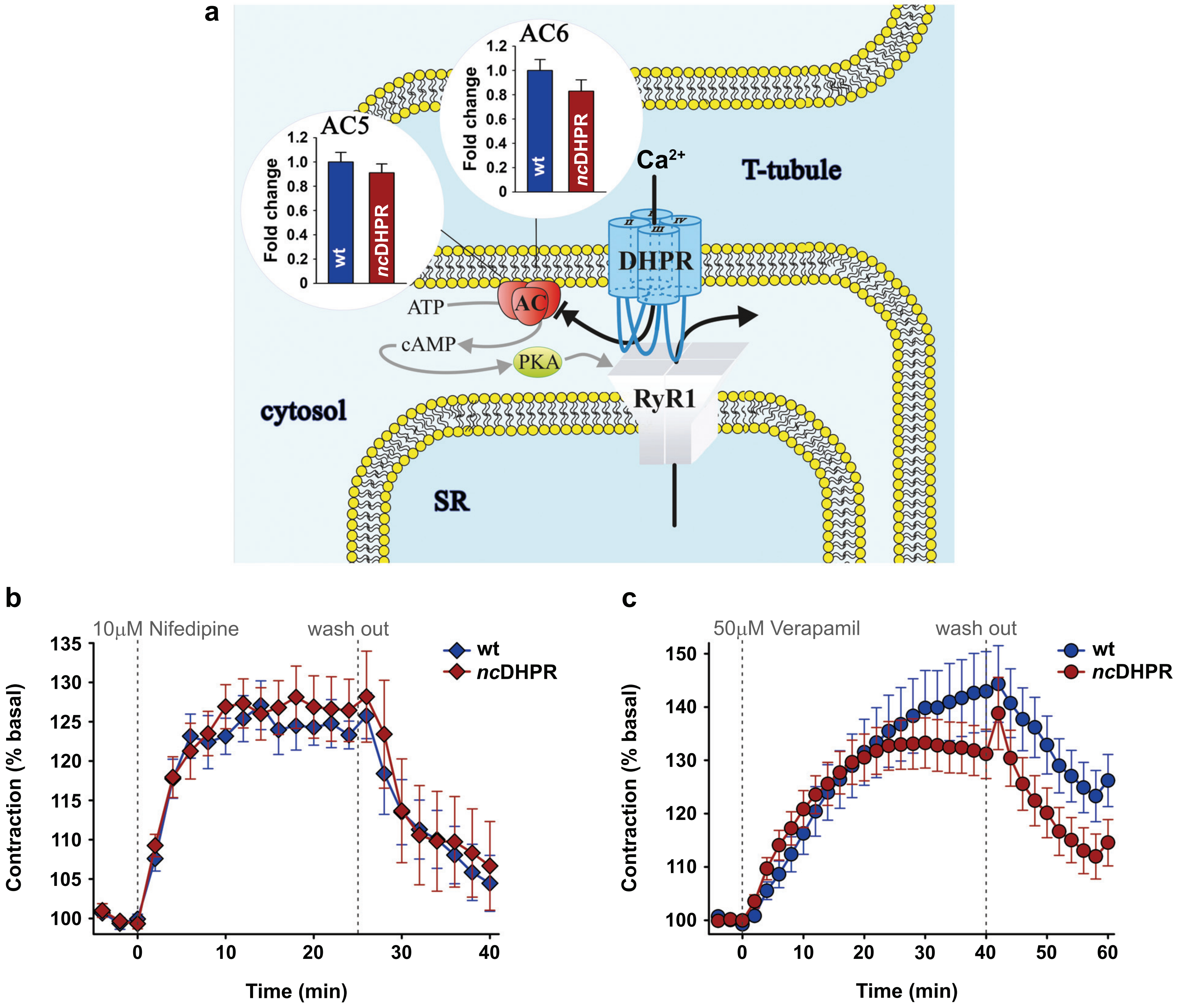

Supplementary Figure 13 | No adaptive transcriptional down-regulation of adenylyl cyclase and attenuation of skeletal muscle contraction in the *nc*DHPR mouse model. (**a**) Schematic representation of the skeletal muscle triad with T-tubular invagination (T-tubule) of the sarcolemma adjacent to the sarcoplasmic reticulum Ca^{2+} store (SR). Illustrated is a signalling cascade model proposed by others[29], according to which the DHPR Ca^{2+} influx is thought to attenuate skeletal muscle contraction via inhibition of Ca^{2+}-sensitive adenylyl cyclase (AC) isoforms, AC5 and AC6. Inhibition of ACs causes reduction in cyclic AMP (cAMP) levels which leads to diminished protein kinase A (PKA) activation and finally, results in reduced PKA phosphorylation of RyR1. (**Inset**), Contrary to the hypothesis of the authors[29] and to our expectations based on the fact that intracellular Ca^{2+} release was unaltered in *nc*DHPR mice (see Fig. 1f, Fig. 2 and Supplementary Fig. 2b), TaqMan® qRT-PCR assay (comparative CT method) with *EEF1A2* as reference gene, did not show compensatory transcriptional down-regulation of AC5 and AC6 in skeletal muscle of neonatal *nc*DHPR (n=18) compared to (P>0.05) wt (n=18) mice. Likewise, comparable results were obtained from adult SOL and EDL muscles (Supplementary Fig. 10). (**b,c**) *Ex vivo* isometric contraction measurements (twitch stimulus: 0.33 Hz, 2 ms, 25 V) on adult diaphragm muscles after perfusion with (**b**) 10 µM nifedipine or (**c**) 50 µM verapamil revealed no differences (P>0.05) in the amplitude or kinetic of the Ca^{2+} antagonist-dependent amplification of muscle contraction between *nc*DHPR (maximum twitch force (as % of basal values) =128.18±5.79; n=6 and 138.79±6.78; n=7, respectively) and wt (125.76±2.90; n=7 and 144.31±7.19; n=7, respectively) mice. Dotted line at time point zero indicates application of the Ca^{2+} antagonist after a short acclimatisation phase of the diaphragm muscle in Ringer solution and the second dotted line indicates "wash out" of the Ca^{2+} antagonist with antagonist-free Ringer solution. All recordings were performed at room temperature (~26 °C). Data are represented as mean±s.e.m.; P determined by unpaired Student's t-test.

Supplementary Table 1.

TaqMan® RT-PCR assay of key triadic proteins involved in EC coupling and Ca^{2+} homeostasis in skeletal muscle of neonatal mice with *ACTB* as the reference gene, revealed no change between *nc*DHPR and wt mice.

All PCRs were performed in triplicates on 18 first-strand replicates from 9 pups.

**Target**	**Mean ± SEM fold change**	
	**wt**	***nc*DHPR**
**AC5**	1.00 ± 0.04	1.10 ± 0.11
**AC6**	1.00 ± 0.05	0.88 ± 0.04
**CSQ1,2**	1.00 ± 0.09	0.89 ± 0.07
**DHPR**	1.00 ± 0.07	0.91 ± 0.05
**RyR1**	1.00 ± 0.04	0.93 ± 0.07
**NCX1**	1.00 ± 0.07	0.93 ± 0.04
**NCX3**	1.00 ± 0.32	0.92 ± 0.22

**Target**	**Mean ± SEM fold change**	
	**wt**	***nc*DHPR**
**Orai1**	1.00 ± 0.06	0.97 ± 0.03
**Orai2**	1.00 ± 0.06	0.91 ± 0.05
**Orai3**	1.00 ± 0.04	0.96 ± 0.07
**PMCA1**	1.00 ± 0.10	0.89 ± 0.05
**SERCA1**	1.00 ± 0.09	0.95 ± 0.05
**STIM1**	1.00 ± 0.07	0.97 ± 0.06
**TRPC1**	1.00 ± 0.07	0.89 ± 0.05

Supplementary Table 2.

Sequences of primers and probes used in quantitative TaqMan® RT-PCR assay of key triadic proteins involved in EC coupling and Ca^{2+} homeostasis.

Target	Forward primer (5' - 3')	Reverse primer (5' - 3')	FAM - Probe - BHQ1 (5' - 3')
**AC5**	CACCCTGGTGTTCCTCTCGG	GTGCTCCTCTGCCAGGCAGCC	GAGTTGCACATGAACATGT
**AC6**	TCTGCTTGTGTTCATCTCTG	CAGCATCCGGGCCGCGCAGGT	TGATTACAGGTAAACATGT
**CSQ1**	CCTTTGCAGAGGAAGCAGATCC	GAGGTCGGGGTTCTCAGTGTTG	CTCTARGAACTCATAGCCATC
**CSQ2**	CCTTTGCGGAGAAGAGTGACCC	CTCAAGTCAGGATTGTCAGTGTTG	CTCTARGAACTCATAGCCATC
**DHPR**	AACCTGGTGCTGGGTGTCCTG	TCTCTCGGAGCTTTTGGAAGGTTC	CTCTTTGGTGAATTCTCCAC
**NCX1**	GTTTGTTGCTCTTGGAACCTCGGTG	GTGACATTGCCTATAGACGCATCTG	TTTGCTGGCAAATGTRTCTG
**NCX3**	TTTTGTGGCATTCGGCACCTCTGTG	GTGACGTTGCCAATGGAAGCATCTG	TTTGCTGGCAAATGTRTCTG
**Orai1**	CTCGGCTCTGCTCTCCGGCTTC	TGAGCAACCCTGGTGGGTAGTCATG	TCCACCATCGCTACCATGG
**Orai2**	CCTCAGCCCTCCTGTCTGGCTTC	AGCAGGGGCTGAGGGTACTGGTAC	TCCACCATGGCCACCATGG
**Orai3**	CATCTGCTCTGCTGTCGGGCTTC	CCACCAGCAGGCCTGGTGGGTAT	TCCACCATGGCCACCATGG
**PMCA1**	TTATCAACCTCCGGAAGGGGATAAT	GCTCCTTCAATCCACCCCGTTTCT	GAAACTTCTCCACAAAGTGC
**RyR1**	CAGTGGACTACCTCCTGCGGC	GTTTCTCTTTCCCTGTTCCTCGATG	AGTCACTGATGGATTCCTGC
**SERCA1**	CCCTCACCACCAACCAGATGTCAGTT	CAGTGATGGAGAACTCGTTCAGTGAGC	CTTGTCAATGATGAACATCTTGC
**STIM1**	GATGATGTGGATCATAAAATCCTAAC	ATCTGCTGCCACCGGTGCA	ACTCAGAGCTTGCTTAGC
**TRPC1**	GTCTGAAACTTGCTATCAAATATAACCAG	CGGTAACCTGACATCTGTCCAAAC	TTGACTGGGAGACAAACTCC
ACTB	CTGAACCCTAAGGCCAACCGTGA	GCCTGGATGGCTACGTACAT	CATGATCTGGGTCATCTTT
EEF1A2	GTGACAACATGCTGGAGCCTTC	GACACGCCGCTTGCATTTCCTT	TTGAACCATGGCATATTAGG

Supplementary Table 3.

Antibodies used in western blotting.

Antigen	Host species and type	Dilution and blocking buffer	Reference
**DHPR**	Mouse, mono	1:400; 5% skim milk in TBST	(39)
**RyR (Pan)**	Mouse, mono	1:40; 5% skim milk in TBST	(39)
**STIM1 (C-terminal)**	Rabbit, poly	1:1,000; 5% skim milk in TBST	(39)
**TRPC1**	Mouse, mono	1:500; 5% skim milk in TBST	Santa Cruz
**Orai1 (N-terminal)**	Rabbit, poly	1:200; 5% BSA in TBST	Gift from Prof. V. Flockerzi
**CSQ (Pan)**	Mouse, mono	1:1,000; 5% skim milk in TBST	(39)
**SERCA (Pan)**	Rabbit, poly	1:2,000; 5% skim milk in TBST	(39)
**PMCA1**	Rabbit, mono	1:1,000; 5% skim milk in TBST	Abcam
**NCX1**	Rabbit, poly	1:500; 5% BSA in TBST	Abcam
**GAPDH**	Mouse, mono	1:50,000; 5% skim milk in TBST	(39)

**Anti-rabbit IgG, IRDye® 680RD**	Goat	1:10,000	LI-COR, Inc.
**Anti-mouse IgG, IRDye® 800CW**	Goat	1:10,000	LI-COR, Inc.
